# X-ray crystal structure of proliferating cell nuclear antigen 1 from *Aeropyrum pernix*

**DOI:** 10.1107/S2053230X24009518

**Published:** 2024-10-09

**Authors:** Takahiro Yamauchi, Makiko Kikuchi, Yasuhito Iizuka, Masaru Tsunoda

**Affiliations:** a Graduate School of Life Science and Technology, Iryo Sosei University, Iwaki, Fukushima, Japan; b Department of Pharmacy Fukushima Rosai Hospital Iwaki Fukushima Japan; c Graduate School of Science and Engineering, Iryo Sosei University, Iwaki, Fukushima, Japan; d Faculty of Pharmacy, Iryo Sosei University, Iwaki, Fukushima, Japan; Osaka University, Japan

**Keywords:** proliferating cell nuclear antigens, DNA replication, *Aeropyrum pernix*, amino-acid-rich medium, lactose operon

## Abstract

Proliferating cell nuclear antigen (PCNA) plays a critical role in DNA replication by enhancing the activity of various proteins involved in replication. In this study, the crystal structure of ApePCNA1, one of three PCNAs from the thermophilic archaeon *Aeropyrum pernix*, was elucidated. ApePCNA1 was cloned and expressed in *Escherichia coli* and the protein was purified and crystallized. The resulting crystal structure determined at 2.00 Å resolution revealed that ApePCNA1 does not form a trimeric ring, unlike PCNAs from other domains of life. It has unique structural features, including a long interdomain-connecting loop and a PIP-box-like sequence at the N-terminus, indicating potential interactions with other proteins. These findings provide insights into the functional mechanisms of PCNAs in archaea and their evolutionary conservation across different domains of life. A modified medium and protocol were used to express recombinant protein containing the *lac* operon. The expression of the target protein increased and the total incubation time decreased when using this system compared with those of previous expression protocols.

## Abstract 2

The crystal structure of proliferating cell nuclear antigen 1 from the thermophilic archaeon *A. pernix* was determined at 2.00 Å resolution. This crystal structure revealed unique features in the N-terminus, including a PIP-box-like sequence, suggesting potential interactions with other proliferating cell nuclear antigens.

## 1. Introduction

DNA replication is a key event in the cell cycle that precedes cell division. It involves various proteins, including DNA-dependent DNA polymerases (Yao & O’Donnell, 2016 ▸). Among these factors, proliferating cell nuclear antigen (PCNA) forms a trimeric ring (sliding clamp) around DNA and directly enhances the activity of proteins involved in replication (Maga & Hübscher, 2003 ▸). Several enzymes bind to PCNA via the PCNA-interacting peptide-box (PIP-box) motif, including DNA-dependent DNA polymerases, flap endonucleases and DNA ligases involved in DNA replication (MacNeill, 2016 ▸; Moldovan *et al.*, 2007 ▸; Winter & Bunting, 2012 ▸). PCNA has a PIP-box binding site near the C-terminus that is oriented in the direction of DNA replication (Horsfall *et al.*, 2021 ▸). Sliding clamps can be considered to be DNA scaffolds that allow specific enzymes (such as DNA polymerase) to bind and catalyze DNA replication.

Sliding clamps are involved in cell proliferation and are frequently expressed in cancer cells (Chen *et al.*, 2021 ▸). They have attracted attention as a potential target for disease diagnosis and treatment (Wendel *et al.*, 2023 ▸). Furthermore, bacterial β-subunit (a monomer of the bacterial sliding-clamp complex) inhibitors have been explored to develop new antimicrobial agents (Liu *et al.*, 2021 ▸; Nedal *et al.*, 2020 ▸; Pandey *et al.*, 2017 ▸). The sliding clamp is one of the earliest reported proteins to have its three-dimensional structure determined, and has been described in many species, including humans, eukaryotes and bacteria (Georgescu *et al.*, 2008 ▸; Gulbis *et al.*, 1996 ▸; Williams *et al.*, 2006 ▸). The sliding clamp is widely present in the three major domains of living organisms, forming a β-clamp dimer in bacteria and a PCNA trimer in eukaryotes. In these two domains, a single PCNA or β-clamp gene is present in the genome, and the sliding clamp refers to a homo-complex. In the archaeal domain, Euryarchaeota form homotrimers encoded by a single gene, whereas some species belonging to the Crenarchaeota family have multiple PCNAs that form heterotrimers and homotrimers (MacNeill, 2016 ▸).

*Aeropyrum pernix* is a crenarchaeon that inhabits high-temperature and aerobic conditions and was discovered on Kodakara-Jima Island, Japan (Sako *et al.*, 1996 ▸). Analysis of the *A. pernix* genome showed that the sequence contains three PCNA genes (Kawarabayasi *et al.*, 1999 ▸): ApePCNA1 (*APE_0162*), ApePCNA2 (*APE_0441.1*) and ApePCNA3 (*APE_2182*) in the KEGG database (Kanehisa *et al.*, 2017 ▸). Of the *A. pernix* genes, approximately 30% have an ATG start codon, approximately 50% have a TTG start codon and 20% have a GTG start codon (Yamazaki *et al.*, 2006 ▸). The start codon of the APE_0162 gene is GTG, and the molecular weights of the recombinant and native (cell-extract) proteins are identical on sodium dodecyl sulfate–polyacrylamide gel electrophoresis (SDS–PAGE; Daimon *et al.*, 2002 ▸). The sliding clamp of *A. pernix* forms heterotrimers and enhances polymerase activity in the ApePCNA1–ApePCNA2–ApePCNA3 and ApePCNA2–ApePCNA3 complexes (Imamura *et al.*, 2007 ▸). ApePCNA1 is expressed in *A. pernix* cells but is not essential for its *in vitro* activity. However, the specific activity of ApePCNA1 has not been determined, and its three-dimensional structure has not yet been analyzed. In this study, we determined the crystal structure of ApePCNA1 and described its features.

## 2. Materials and methods

### 2.1. Macromolecule production

The *APE_0162* gene encoding ApePCNA1 from *A. pernix* K1^T^ was amplified from the genomic DNA of strain NBRC 100138^T^ (obtained from NBRC, NITE, Kisarazu, Japan) using polymerase chain reaction (PCR). The PCR product was treated with the NdeI and BglII restriction enzymes and was subcloned into pET-11a vector (Merck KGaA, Darmstadt, Germany). The plasmid was introduced into *Escherichia coli* Rosetta-gami (DE3) cells by electroporation on ice for 4 ms at 1.80 kV and 25 µF; the cells were incubated in Super Optimal broth with catabolite repression at 310 K for 30 min and spread onto Luria–Bertani (LB) agar containing ampicillin and chloramphenicol. A colony was selected and grown as a primary culture in 15 ml LB broth (Lennox) containing 50 µg ml^−1^ ampicillin and 34 µg ml^−1^ chloramphenicol for 9 h at 310 K. Subsequently, 7.5 ml bacterial suspension from the primary culture was grown in 1 l 1% Bacto tryptone (Becton, Dickinson & Co., Franklin Lakes, New Jersey, USA), 0.5% peptone (Kyokuto Pharmaceutical Industrial, Tokyo, Japan), 0.5% sodium chloride, 50 µg ml^−1^ ampicillin and 34 µg ml^−1^ chloramphenicol for 12 h at 310 K. The cells were cultured to an *A*_600_ of 0.6, and expression of the *APE_0162* gene was induced by adding 10% lactose to a final concentration of 0.4%. Cultivation continued for a further 4 h at 310 K.

The cells were harvested, resuspended in 20 m*M* Tris–HCl pH 8.0, 50 m*M* NaCl, disrupted by sonication and heated for 30 min at 353 K. Heat-resistant fractions were obtained by centrifugation (8000*g* for 15 min at 277 K). Soluble proteins were precipitated using 40% saturated ammonium sulfate. The precipitate was resuspended in 10 ml 20 m*M* Tris–HCl pH 8.0 and dialyzed three times against 1 l 20 m*M* Tris–HCl pH 8.0 containing 200 m*M* NaCl (the three dialysis times were 30 min, 1 h and overnight). The dialysate was loaded onto a HiTrap Q HP column (5 ml; Cytiva, Marlborough, Massachusetts, USA) and ApePCNA1 protein was eluted with 200–500 m*M* sodium chloride.

The eluted protein was concentrated to 100 µl by ultrafiltration using an Amicon Ultra-15 10K centrifugal filter (Merck) and applied onto a HiLoad 16/600 Superdex 75 pg column (Cytiva) pre-equilibrated with 20 m*M* Tris–HCl pH 8.0, 200 m*M* sodium chloride. The macromolecule-production process is summarized in Table 1 ▸.

### 2.2. Crystallization

Purified ApePCNA1 was concentrated to 10 mg ml^−1^ via ultrafiltration using an Amicon Ultra 10K centrifugal filter. The buffer solution was changed to 20 m*M* Tris–HCl pH 8.0 at the same time. Crystallization was performed by hanging-drop vapor diffusion in a VDX Plate with sealant (Hampton Research, Aliso Viejo, California, USA) at 298 K. The initial crystallization conditions were screened using Wizard Classics 1 and 2 (Rigaku Reagents, The Woodlands, Texas, USA) and Index (Hampton Research). Finally, colorless, origami shuriken-shaped (paper ninja star-like) crystals grew after two days in 100 m*M* McIlvaine buffer pH 4.2–4.6, 100–250 m*M* lithium sulfate, 7% propan-2-ol. The crystallization conditions are summarized in Table 2 ▸.

### 2.3. Data collection and processing

The ApePCNA1 crystals were collected and packed into a Universal V1-Puck at 77 K in liquid nitrogen. Diffraction data for ApePCNA1 were collected on BL-1A at the Photon Factory, Tsukuba, Japan. The diffraction images were processed (indexed, integrated, scaled and merged) using *XDSGUI* (Brehm *et al.*, 2023 ▸). The data-collection statistics are summarized in Table 3 ▸.

### 2.4. Structure solution and refinement

Phases were determined by molecular replacement using *MOLREP* (Vagin & Teplyakov, 2010 ▸) and the PCNA model from *Pyrococcus furiosus* (PDB entry 1iz4; Matsumiya *et al.*, 2001 ▸). The model was rebuilt using *ARP*/*wARP Classic* (Langer *et al.*, 2008 ▸) and 92 water molecules were added. The structure was modified using *Coot* (Emsley *et al.*, 2010 ▸) and refined using *REFMAC*5 (Murshudov *et al.*, 2011 ▸). These processes were performed within the *CCP*4*i* interface (Potterton *et al.*, 2003 ▸) using the *CCP*4 suite (Agirre *et al.*, 2023 ▸). The ApePCNA1 structure was validated using *Coot* and the wwPDB validation services (Young *et al.*, 2017 ▸) during deposition. Figures were constructed using *PyMOL* (https://www.pymol.org/). The refinement statistics are summarized in Table 4 ▸.

## 3. Results and discussion

### 3.1. Sequence comparison of PCNAs

**Figure 1:**
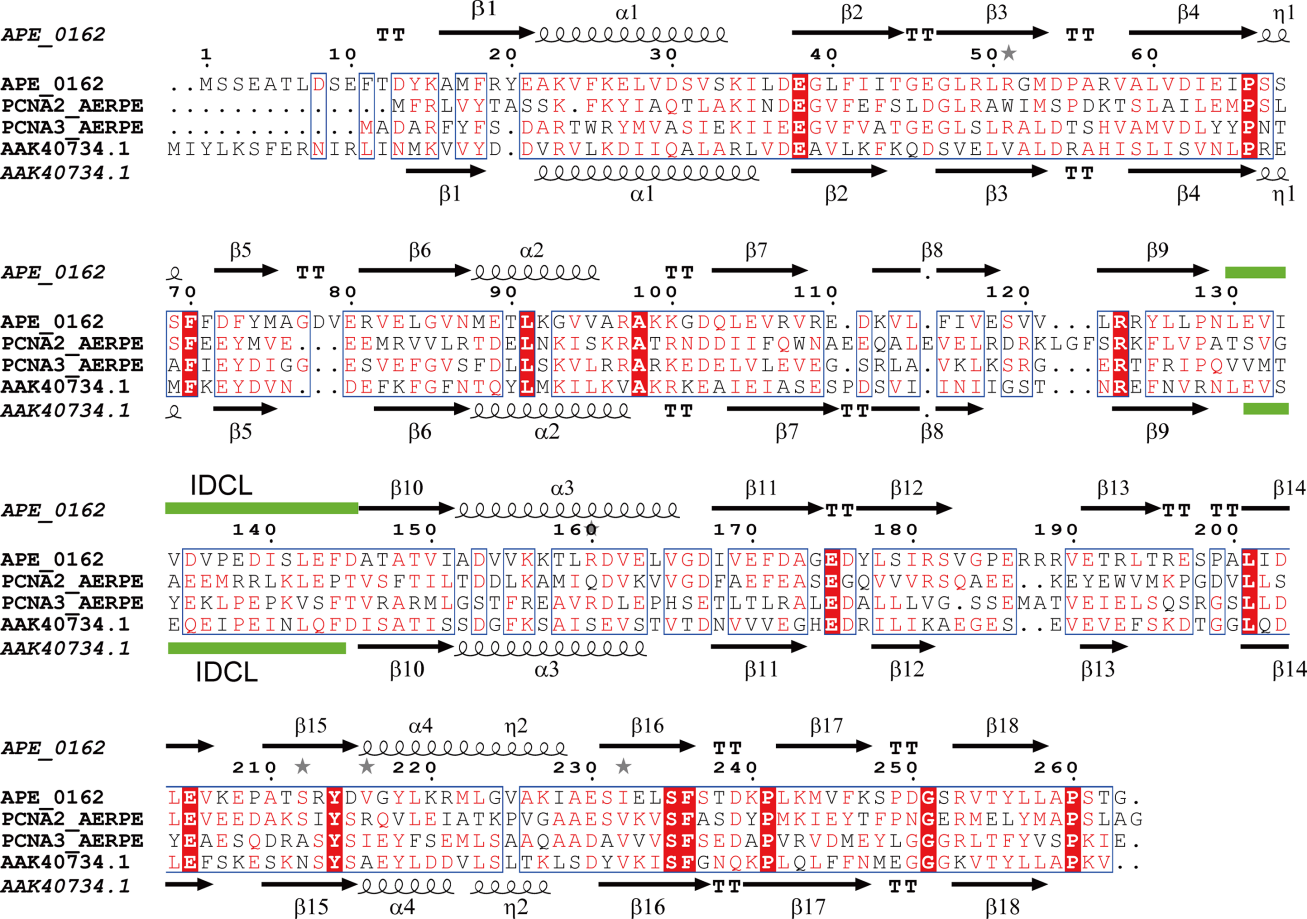
Multiple sequence alignment of ApePCNAs and SsoPCNA3. Helices, β-sheets and the IDCL are represented by coils, arrows and green bars, respectively. Columns with residues that are more than 70% similar according to physicochemical properties (threshold set to 0.7) are framed in blue and amino-acid residues with 100% identity are highlighted by a red background. This figure was created using *ESPript* 3.0 (Robert & Gouet, 2014 ▸).

The amino-acid sequence identities between ApePCNA1 and ApePCNA2, between ApePCNA2 and ApePCNA3 and between ApePCNA3 and ApePCNA1 were 28.16%, 21.81% and 28.57%, respectively. The average identity among the three PCNAs was 26.18% (Fig. 1 ▸). The ApePCNA1 sequence was approximately ten residues longer at the N-terminus than the other two sequences. Similarly, PCNAs from *Saccharo­lobus solfataricus* contained a long N-terminal region, as in SsoPCNA3 (PDB entry 2ix2, chain *C*; protein ID AAK40734.1; Williams *et al.*, 2006 ▸). However, the homology between ApePCNA1 and SsoPCNA3 (24.9%) was lower than those between the ApePCNAs, and only Met1 matched the N-terminal region before the second methionine residue of each PCNA (Met17 in ApePCNA1 and Met16 in SsoPCNA3). These long N-terminal amino-acid sequences (ApePCNA1, Q9YFT8; SsoPCNA3, P57765) are considered to be errors in the initial positions in the UniProt database (The UniProt Consortium, 2023 ▸). However, as discussed later, they can be used as anchor sequences.

### 3.2. Evaluating the enhanced expression system

**Figure 2:**
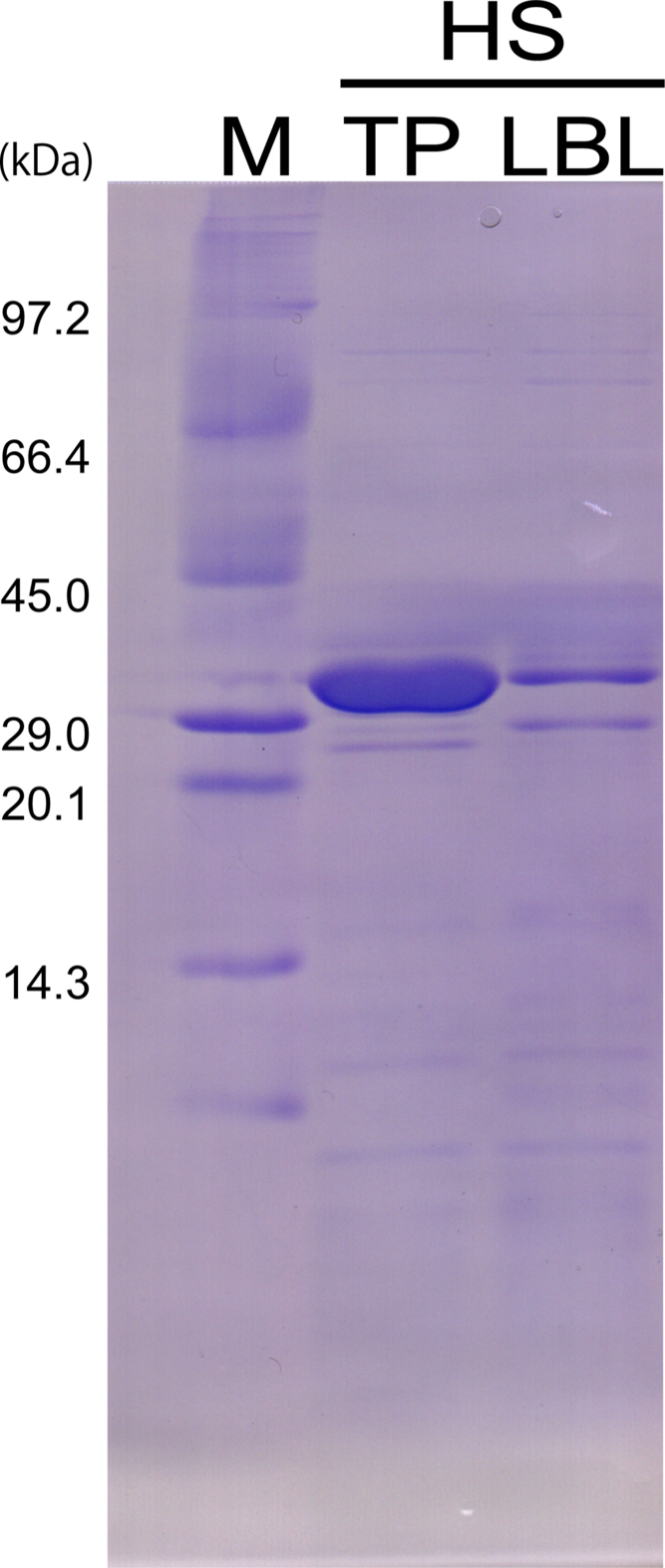
Comparison of ApePCNA1 expression between the two media. In the SDS–PAGE image, the molecular-weight marker (lane M) is on the left, TP medium (lane TP) is in the middle and Lennox LB medium (lane LBL) is on the right. Supernatants (lanes HS) of each sample with the same weight were compared after heat treatment.

ApePCNA1 was effectively expressed in an amino-acid-rich medium that differs from the Lennox LB medium (Lennox, 1955 ▸) that is widely used in protein-expression systems. SDS–PAGE showed that the expression in 1% tryptone, 0.5% peptone, 0.5% NaCl broth (TP broth) was higher than that in Lennox LB medium (Fig. 2 ▸). Furthermore, the expression of *E. coli*-derived proteins (other than the target proteins) was significantly lower in this medium than in conventional LB medium. This medium is mostly composed of amino acids, which limit energy and nucleic acid metabolism. For example, the expression of these metabolism-related proteins is reduced owing to the extremely low amount of nucleic acids in the medium, since the *de novo* pathway is dominant in nucleic acid metabolism and the salvage pathway is of little significance. Therefore, the specificity of the expression levels of the target proteins may be attributed to the reduced expression of proteins required for their metabolism. We examined the timing of lactose addition by inducing protein expression using the *lac* operon. Considering the simplicity of this technique and the risk of contamination during incubation, it is preferable to mix lactose with the culture medium and sterilize it at the time of use, rather than separately preparing and sterilizing the culture medium and lactose solution before mixing them. However, the growth rate of the host *E. coli* was suppressed when ApePCNA1 was overexpressed in a medium containing lactose, and the incubation time required for the solution to reach visually observable turbidity doubled to 26 h. Thus, the premix protocol was unsuitable for *E. coli* growth. To resolve this issue, lactose was added after *E. coli* reached an *A*_600_ of 0.6, which facilitated the successful growth of *E. coli* cells overexpressing ApePCNA1.

### 3.3. Crystal structure of ApePCNA1

**Figure 3:**
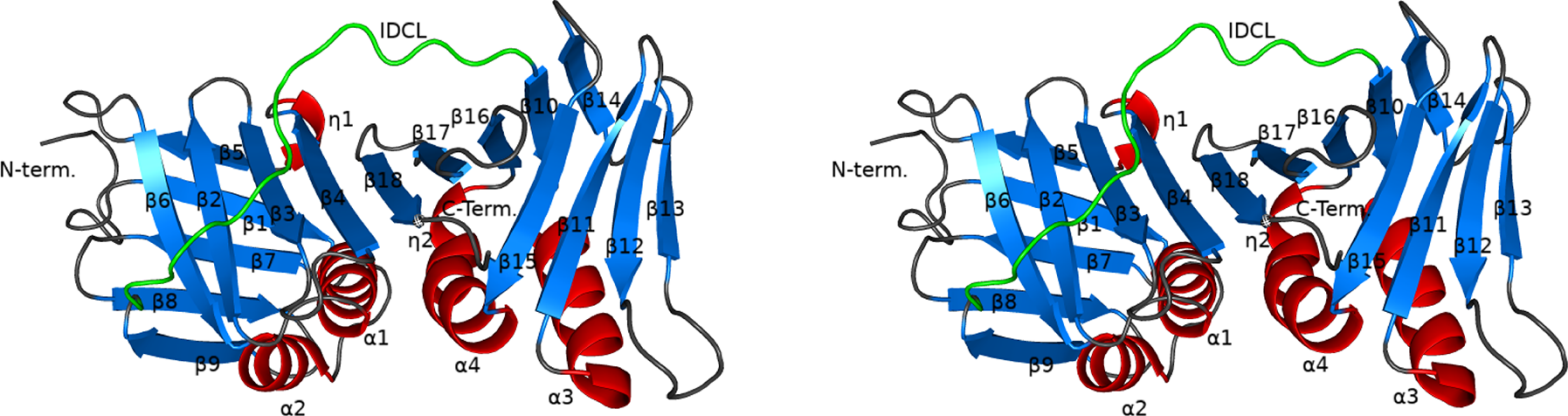
Stereoview of the ApePCNA1 structure. The protein is depicted as a ribbon diagram, with the α-helices, β-sheets, loops and IDCL colored red, marine blue, gray and green, respectively.

**Figure 4:**
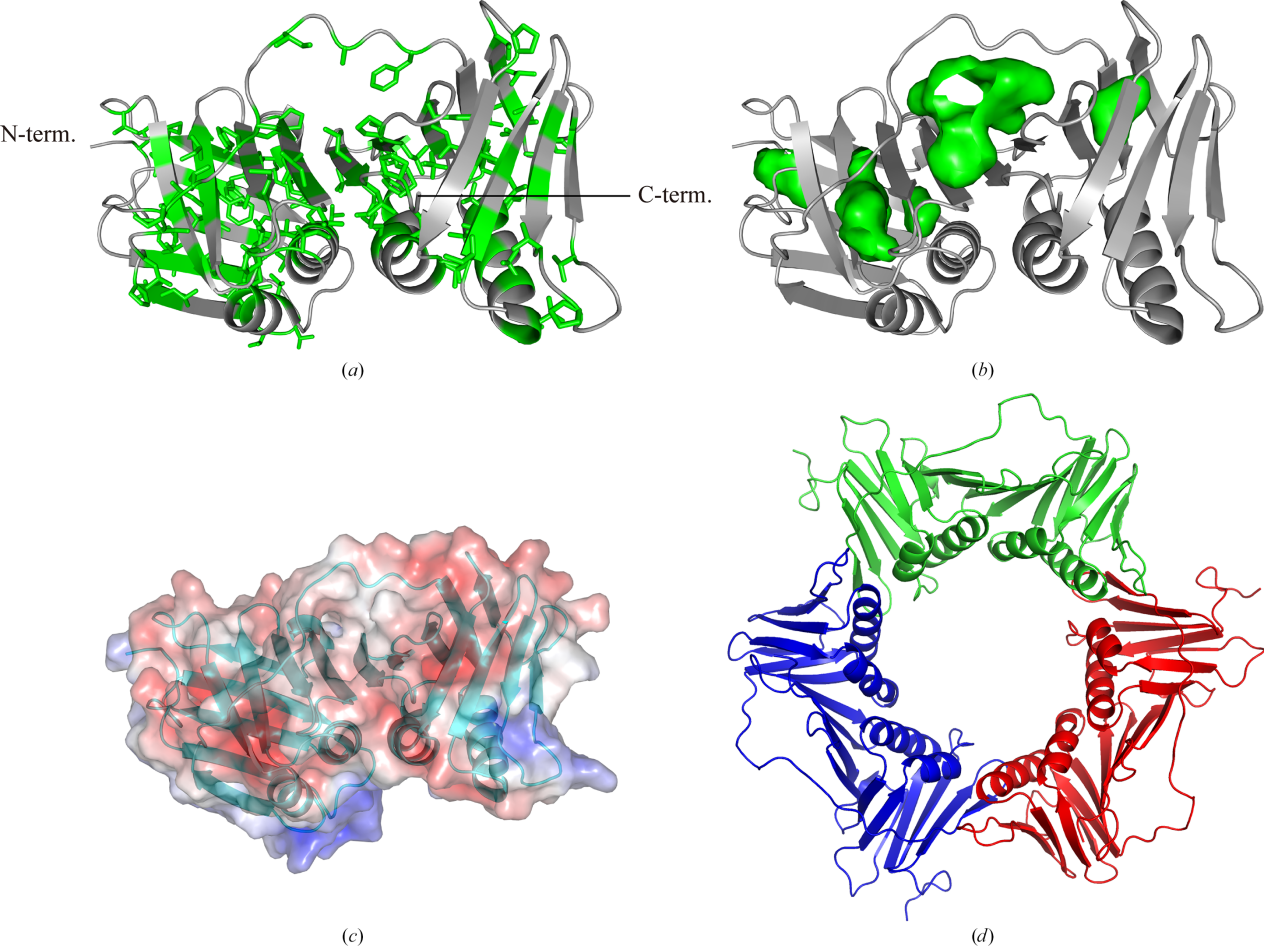
Structural features of ApePCNA1. (*a*) Hydrophobic amino acids are shown as green sticks. (*b*) Cavity regions are shown as green meshes. The cavities were drawn using *PyMOL*. (*c*) Electrostatic potentials on the molecular surface are shown in blue and red for positive and negative charge, respectively. (*a*), (*b*) and (*c*) are viewed from the same orientation as in Fig. 3 ▸. (*d*) Model of the ApePCNA1 homotrimeric ring viewed from the threefold axis direction. Each subunit is colored differently.

ApePCNA1 crystals diffracted to a maximum of 1.59 Å resolution and the final structure was determined at a resolution of 2.00 Å (Fig. 3 ▸). The final resolution cutoff was 2.0 Å, because the *R*_marge_ and *I*/σ(*I*) showed abnormal values in the resolution range 1.96–1.66 Å, although the statistics for the outer shell (resolution range 1.66–1.59 Å) were normal. Most amino-acid residues of ApePCNA1 (except for the N-terminal Met1-Ser2-Ser3-Glu4 and the C-terminal Gly263) were assigned in the final structure. The crystal structure contains a single ApePCNA1 subunit within the asymmetric unit. The crystals did not contain a threefold axis and the subunits did not form a ring-shaped trimeric structure. The ApePCNA1 subunit is composed of N- and C-terminal domains and a long loop called the inter-domain-connecting loop (IDCL) that connects the two domains formed by Leu130–Gln145. The N- and C-terminal domains consist of two β-sheets and two α-helices. In the N-terminal domains, each β-strand (β5, β1, β7, β8, β9 and β6, β2, β3, β4) forms small β-sheets. In the C-terminal domains, β15, β11, β12, β13 and β14, β10, β16, β17, β18 form β-sheets. One of the two β-sheets in each domain is linked to a β-sheet in the neighboring domain in the same molecule to form a large continuous β-sheet (β6, β2, β3, β4, β18, β17, β16, β10, β14). Therefore, the ApePCNA1 subunit contains one large β-sheet and two small β-sheets. All of these β-sheets are arranged in an antiparallel manner. The four α-helices are arranged in an inverse parallel manner and are positively charged. Of these, η2 follows α4 to form a tighter helix. The individual β-strands that make up the large β-sheet are aligned parallel to the antiparallel α-helices, but although the middle of the sheet is close, the edges of the sheet are further away from the α-helices. The two small β-sheets that do not belong to the large β-sheet are arranged so that one covers one end of the large β-sheet and the other covers one end of the α-helices. The inner side of the ApePCNA1 subunit surrounded by these secondary structures is rich in hydrophobic residues that form a series of hydrophobic inter­actions (Fig. 4 ▸*a*). Cavity regions are present near the contacts between the two sides and the dorsal β-sheet (Fig. 4 ▸*b*). A cavity is also present between the C-terminal side of the IDCL region and the back β-sheet, which is rich in hydrophobic residues. The overall structure is similar to those of the homotrimeric ring of human PCNA (PDB entry 1axc; Gulbis *et al.*, 1996 ▸), a monomer of the PCNA ring from yeast (PDB entry 1plq; Krishna *et al.*, 1994 ▸) and a PCNA monomer from the thermophilic archaeon *P. furiosus* (PDB entry 1ge8; Matsumiya *et al.*, 2001 ▸). Structural alignment was performed for these PCNAs (human PCNA, yeast PCNA and *P. furiosus* PCNA). *PyMOL* was used for the alignment, which was performed using C^α^ atoms. The r.m.s.d.s for each were 1.178 Å (the average value for the trimer), 1.061 Å and 0.881 Å, respectively.

### 3.4. Interaction of ApePCNA1 with other molecules

**Figure 5:**
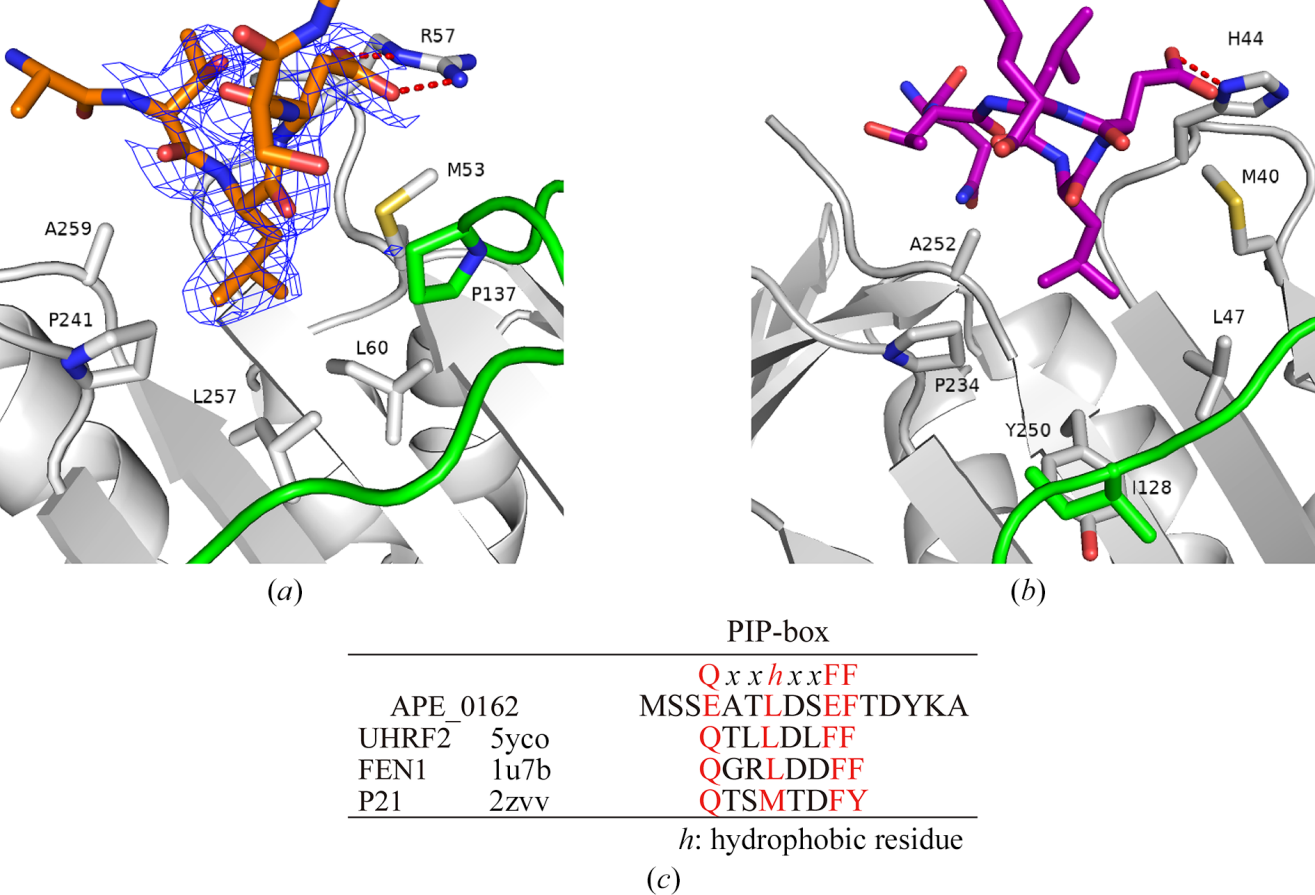
Comparison of the PCNA structures and PIP-box sequences. (*a*) Omit *F*_o_ − *F*_c_ difference map (blue mesh) calculated without the N-terminus of ApePCNA1 (orange) contoured at 2σ. The ICDL is colored green and red dashes represent hydrogen bonds. (*b*) Human PCNA with UHRF2^PIP^ (PDB entry 5yco) is shown in the same orientation as in (*a*). (*c*) The PIP-box consensus sequence was compared with the N-terminal sequence of ApePCNA1 and proteins with the PIP-box sequence [PDB entries 1u7b (Bruning & Shamoo, 2004 ▸) and 2zvv (Strzalka *et al.*, 2009 ▸)].

The PCNA rings from other species have an inward α-helix region (Li *et al.*, 2021 ▸). The positive surface charge of the α-helix region suggests that ApePCNA1 has an α-helix region located in the inner part of the ring that interacts with the negative charge of the DNA double helix like that in other species (Fig. 4 ▸*c*). The trimeric ring model based on the structure of ApePCNA1 is connected by two adjacent small β-sheets between each subunit (Fig. 4 ▸*d*). In addition, the long N-terminus may be disordered in aqueous solution, suggesting that it does not form a homotrimer. This insight follows the results of gel-filtration analysis in a previous study (Imamura *et al.*, 2007 ▸). The IDCL region of PDB-deposited PCNAs is a movable, partially unfolded loop that often has a disordered structure. In contrast, electron density was observed in the IDCL region of ApePCNA1. Contrary to our expectations, clear electron density was observed at the N-terminus of ApePCNA1, which was expected to be a partially unfolded loop owing to its length. These long loops are the contact points of the adjacent subunits related to the screw axis in the space group (*P*4_3_2_1_2) of the crystal. In general, the C-terminal side of the sliding clamp is considered to be the direction of DNA replication, and a group of enzymes responsible for DNA replication bind to the binding pocket near the IDCL (González-Magaña & Blanco, 2020 ▸). Interestingly, the N-terminus of ApePCNA1 interacted with the cleavage groove of the adjacent ApePCNA1 molecule (Fig. 5 ▸*a*). In this interaction, the carboxyl group of the N-terminal Asp8 is connected to Arg57 of the neighboring subunit by a hydrogen bond. Additionally, the NH of the main chain of Leu7 formed a hydrogen bond to the CO of the main chain of Arg57 in the neighboring subunit. The hydrophobic side chain of Leu7 is placed in a hydrophobic pocket formed by Met53, Leu60, Pro137, Pro241, Leu257 and Ala259 in an adjacent subunit. This interaction is like that observed between human PCNA and UHRF2^PIP^ (PDB entry 5yco; Chen *et al.*, 2017 ▸; Fig. 5 ▸*b*). The consensus sequence of the PIP-box is Q*XXhXXaa*, where *X* is any amino acid, *h* is a hydrophobic amino acid and *a* is an aromatic amino acid. The 4-EATLDSEF-11 sequence in the N-terminal region of ApePCNA1 is similar to the consensus sequence of the PIP-box (Fig. 5 ▸*c*). These results suggest that the PIP-box binding site of ApePCNA1 is in a similar location and binds in an analogous manner to those of PCNAs from other species. Furthermore, the N-terminal region of ApePCNA1 was suggested to be an anchor sequence that induces PIP-box binding. These results suggest that ApePCNA1 has an unknown function mediated by an N-terminal anchor sequence that results in PIP-box-like interactions.

In conclusion, we determined the crystal structure of ApePCNA1. A unique interaction via the N-terminus (which contains a PIP-box-like sequence) may be involved in the uncharacterized DNA-replication machinery in thermophilic archaea. We developed a new approach using a modified medium and protocol to express recombinant proteins containing the *lac* operon. These findings may be useful for protein research.

## Supplementary Material

PDB reference: proliferating cell nuclear antigen 1 from *Aeropyrum pernix*, 6aig

## Figures and Tables

**Table 1:** Macromolecule-production information

Source organism	*A. pernix* K1^T^
Expression vector	pET-11a
Expression host	*E. coli* Rosetta-gami (DE3)
Complete amino-acid sequence of the construct produced†	MSSEATLDSEFTDYKAMFRYEAKVFKELVDSVSKILDEGLFIITGEGLRLRGMDPARVALVDIEIPSSSFFDFYMAGDVERVELGVNMETLKGVVARAKKGDQLEVRVREDKVLFIVESVVLRRYLLPNLEVIVDVPEDISLEFDATATVIADVVKKTLRDVELVGDIVEFDAGEDYLSIRSVGPERRRVETRLTRESPALIDLEVKEPATSRYDVGYLKRMLGVAKIAESIELSFSTDKPLKMVFKSPDGSRVTYLLAPSTG

† The hypothetical anchor sequence is underlined.

**Table 2:** Crystallization

Method	Vapor diffusion, hanging drop
Plate type	VDX plate
Temperature (K)	298
Protein concentration (mg ml^−1^)	10
Buffer composition of protein solution	20 m*M* Tris–HCl pH 8.0
Composition of reservoir solution	McIlvaine buffer, lithium sulfate, propan-2-ol
Volume and ratio of drop	4 µl, 1:1
Volume of reservoir (µl)	1000

**Table 3:** Data collection and processing Values in parentheses are for the outer shell.

Diffraction source	Beamline BL-1A, Photon Factory
Wavelength (Å)	1.1000
Temperature (K)	100
Detector	Dectris EIGER X 4M
Crystal-to-detector distance (mm)	92.260
Rotation range per image (°)	1
Total rotation range (°)	180
Exposure time per image (s)	10
Space group	*P*4_3_2_1_2
*a*, *b*, *c* (Å)	69.03, 69.03, 120.86
α, β, γ (°)	90, 90, 90
Mosaicity (°)	0.138
Resolution range (Å)	48.86–2.00 (2.05–2.00)
Total No. of reflections	258157 (12621)
No. of unique reflections	20481 (1056)
Completeness (%)	100.0 (100.0)
Multiplicity	12.5 (13.4)
〈*I*/σ(*I*)〉	34.4 (15.0)
*R*_merge_ †	0.048 (0.164)
*R*_r.i.m._ ‡	0.051 (0.177)
*R*_p.i.m._ §	0.019 (0.067)
Overall *B* factor from Wilson plot (Å^2^)	19.354

† *R*_merge_ = 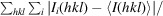

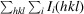
, where *I*(*hkl*) is the intensity of reflection *hkl*.

‡ *R*_r.i.m._ = 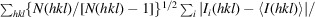

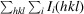
, where *N*(*hkl*) is the data multiplicity.

§ *R*_p.i.m._ = 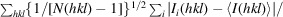

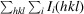
.

**Table 4:** Structure refinement Values in parentheses are for the outer shell.

Resolution range (Å)	48.86–2.00 (2.052–2.000)
Completeness (%)	99.9
σ Cutoff	2.00
No. of reflections, working set	19446 (1403)
No. of reflections, test set	991 (69)
Final *R*_work_†	0.185 (0.196)
Final *R*_free_‡	0.226 (0.251)
Cruickshank DPI	0.1220
No. of non-H atoms	
Protein	1959
Ligand	0
Water	63
Total	2022
R.m.s. deviations	
Bond lengths (Å)	0.014
Angles (°)	1.583
Average *B* factors (Å^2^)	
Protein	29.6
Water	32.6
Ramachandran plot	
Most favored (%)	98.8
Allowed (%)	1.2

† *R*_work_ = 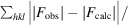

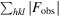
.

‡ *R*_free_ is calculated the same way as *R*_work_ for data omitted from refinement (5% of the reflections in the data set).
